# Mammalian microRNA: an important modulator of host-pathogen interactions in human viral infections

**DOI:** 10.1186/s12929-016-0292-x

**Published:** 2016-10-26

**Authors:** Chet Raj Ojha, Myosotys Rodriguez, Seth M. Dever, Rita Mukhopadhyay, Nazira El-Hage

**Affiliations:** 1 Department of Immunology, Herbert Wertheim College of Medicine, Florida International University, Florida, USA; 2 Department of Cellular Biology and Pharmacology, Herbert Wertheim College of Medicine, Florida International University, Florida, USA

**Keywords:** microRNA, miRISC, Pathogens, Gene regulation

## Abstract

MicroRNAs (miRNAs), which are small non-coding RNAs expressed by almost all metazoans, have key roles in the regulation of cell differentiation, organism development and gene expression. Thousands of miRNAs regulating approximately 60 % of the total human genome have been identified. They regulate genetic expression either by direct cleavage or by translational repression of the target mRNAs recognized through partial complementary base pairing. The active and functional unit of miRNA is its complex with Argonaute proteins known as the microRNA-induced silencing complex (miRISC). De-regulated miRNA expression in the human cell may contribute to a diverse group of disorders including cancer, cardiovascular dysfunctions, liver damage, immunological dysfunction, metabolic syndromes and pathogenic infections. Current day studies have revealed that miRNAs are indeed a pivotal component of host-pathogen interactions and host immune responses toward microorganisms. miRNA is emerging as a tool for genetic study, therapeutic development and diagnosis for human pathogenic infections caused by viruses, bacteria, parasites and fungi. Many pathogens can exploit the host miRNA system for their own benefit such as surviving inside the host cell, replication, pathogenesis and bypassing some host immune barriers, while some express pathogen-encoded miRNA inside the host contributing to their replication, survival and/or latency. In this review, we discuss the role and significance of miRNA in relation to some pathogenic viruses.

## Background

Recent advancements in genomics and proteomics have shown that out of roughly half of the human genome which is transcribed into RNA transcripts, about 2 % is translated into the corresponding amino acid sequences [1]. The remaining 98 % of RNA transcripts are collectively known as non-coding RNAs (ncRNA) which may be divided into small non-coding RNA (sncRNA) or long non-coding RNA (lncRNA) [1, 2]. MicroRNAs (miRNAs) are endogenous small non-coding RNAs regulating gene expression in almost all metazoans [3]. In spite of coding for any proteins, miRNAs carry different information and execute different functions [4]. They regulate gene expression either by complete cleavage or by translational repression of the target mRNAs [3, 5, 6] It has been speculated that approximately 30–60 % of the human coding genome is regulated by thousands of miRNAs with diverse targets [7, 8].

The exciting avenue of miRNA was unraveled in 1993 by the finding that Lin-4, a heterochronic gene previously recognized for its role in regulating the temporal sequence of events involved in *Caenorhabditis elegans* (*C. elegans*) larval development to adult form, regulates the process by synthesizing a pair of small RNAs rather than coding for a protein [9]. Two small Lin-4 RNA transcripts containing complementary sequences to a repeating sequence element within the 3′-untranslated region (3′ UTR) of another mRNA (Lin-14) were identified in *C. elegans* [10]. The finding led to the prediction of a type of RNA-RNA binding and interaction which down-regulates the translation of the target mRNA [4]. Subsequently, the second miRNA (Let-7) with a similar function in the late development of larva was discovered in the same organism [11]. The names for miRNAs are assigned by using the prefix “miR” preceding a unique identification numeric (e.g., miR-1, miR-2 etc.). To make species specific, few letters from the name of the organism are added before miR (e.g.; hsa for *Homo sapiens*, mmu for *mus musculus*, rno from R*attus norvegicus*, ath for *Arabidopsis* plant etc.) [12]. The genes coding for miRNAs are named by capitalization (e.g., MIR-), hyphenation and italicization (e.g., *mir-*) in accordance with the conventions for the particular organism [12, 13]. Currently, 35,828 total and 2588 human encoded mature miRNAs are registered in miRNA database (http://www.mirbase.org/) [14, 15].

With the expanding body of research on miRNAs in relation to important biological processes, their crucial role as regulators of cell differentiation, proliferation and growth, intracellular dynamics, and apoptosis has been established [3, 9]. De-regulated miRNA expression leads to human pathologies including cancer, cardiovascular disease, liver disorders, immunological dysfunction, and metabolic syndromes [5, 9]. There are a growing number of reviews on miRNAs and their role in the aforementioned disorders and some individual pathogenic infections. With this review we present the current understanding of miRNAs and their role in some viral pathogens.

## Biosynthesis and mode of action of miRNA

**Fig. 1:**
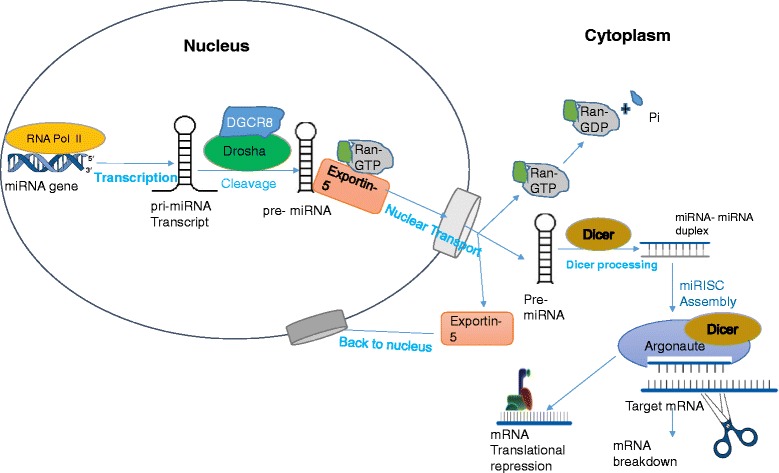
Biosynthesis, processing and effector action of miRNA. miRNA gene is transcribed by RNA polymerase II into a long transcript known as primary miRNA (pri-miRNA) which is further trimmed by microprocessor complex (Drosha and DGCR8) into an approximately 70 nucleotide- long hairpin structure known as pre-miRNA. Subsequently, Exportin-5 binds to the pre-miRNA and to a GTP-Ran, forming a heterotrimeric complex which passes through the nuclear membrane. After translocation to the cytoplasm, the GTPase activity of Ran catalyzes the hydrolysis of GTP into GDP to facilitate the release of pre-miRNA from exportin-5. Exportin-5 then returns to the nucleus and available for another round of pre-miRNA transport. Once released in the cytoplasm, a specialized multi-domain ribonuclease III enzyme known as Dicer further processes the pre-miRNAs to form a miRNA-miRNA duplex. One strand (anti-sense strand) of the resulting miRNA-miRNA duplex is loaded onto Argonaute proteins leading to the formation of miRNA-induced silencing complex (miRISC). Partial complementary base pairing occurs between the seed region (2 to 8 nucleotides from the 5′-proximal region) of the miRNA and the seed map site (complementary to the seed region) of the target mRNA. The ultimate effect may be either endo-nucleolytic cleavage or translational repression of the target mRNA

Genes encoding several identical or similar miRNAs are generally located as a cluster in the genome, where they might be expressed simultaneously [16] or individually depending on the tissue types [17]. In mammals, RNA polymerase II transcribes the gene into a long transcript known as a primary miRNA (pri-miRNA) consisting of single or multiple hairpin structures (Fig. 1) [13, 18–21]. Pri-miRNA is trimmed into an approximately 70 nucleotide long hairpin structure known as pre-miRNA by Drosha complex [22, 23]. The resulting pre-miRNA with a 5′ phosphate overhang and a 2 nucleotide 3′ overhang is recognized by exportin-5 [24] and is transported out of the nucleus [18, 25, 26]. Once released in the cytoplasm, a specialized multi-domain ribonuclease III enzyme known as Dicer excises the pre-miRNA to remove the loop structure leaving the remaining miRNA duplex with a 2 nucleotide 3′ overhang [18, 27, 28]. Some functional miRNA such as miRtrons and Simtrons do not undergo the canonical process for their maturations [29, 30]. In miRtron biogenesis, Drosha cleavage is substituted by splicing of intronic hairpin structures, which is followed by maturation through dicing [31]. Simtron is synthesized by a pathway that involves only Drosha but does not require DGCR8, Dicer, Ago2 or XPO5 for its further processing [32]. After Dicer cleavage and unwinding of the two strands of miR-miR duplex, one strand (anti-sense strand) of the resulting miRNA-miRNA duplex is loaded onto Argonaute proteins and miRNA-induced silencing complex (miRISC) is generated [3, 33].

miRNA regulates gene expression typically targeting and binding to the seed map site in 3′-untranslated region (3′-UTR) of protein coding target messenger RNA (mRNA) leading to degradation or translational repression of the gene [18, 34]. However, miRNAs can also target to the sites other than 3′UTR, such as 5′UTR and the coding regions of mRNA and lead to translational repression [35, 36]. In mammal, perfect complimentary affects the stability and triggers tailing and 3′-to-5′ trimming of miRNA [37]. It has been reported that some miRNAs can also mediate up-regulation of genes and that the genetic down-regulation mediated by miRNA can be reversible [38]. The upregulating miRNAs most likely direct the association of regulatory proteins complexes (Argonaute protein and fragile X mental retardation-related protein 1 (FXR1)) with AU-rich elements (AREs) in the 3′UTR of the mRNA, leading to activation of AREs as a translation signal [39]. Moreover, interaction of miRNA at 5′UTR can also trigger activation of translation [35].

## Role of miRNA in viral infections

miRNAs play a crucial role in mounting an immune response against microbial infections caused by viruses, bacteria, parasites and fungi [40]. Nevertheless, many microorganisms have been shown to modulate the expression of several host miRNAs either to facilitate their own replication, survival and pathogenesis or for some unknown functions [30, 41–43]. Restriction of viral replication by RNA interference (RNAi) either by miRNA or siRNA in human cells is still a controversial issue [44]. Most of the human viruses when in their acute and replicative stages are thought to be resistant to endogenous RNAi mediated by miRNAs [44]. However, strong evidences to prove the role of miRNAs in restricting or promoting the replication and human pathology of viruses such as Hepatitis B and C as well as other viruses have been found [45, 46] Many of the cellular miRNAs affect viral replication either directly by binding to the viral genome or indirectly by targeting host factors related to replication [8].

Virus-encoded miRNAs (vmiRNA) identified in virus-infected human cells and other mammalian hosts significantly influence viral replication and disease progression by modulating viral as well as host cellular mRNA [41, 47]. The first vmiRNA identified in humans was that encoded by the Epstein-Barr virus and subsequently, other members of herpesvirus, polyomavirus and adenovirus families were found to express vmiRNAs [41]. More than 200 vmiRNAs have been reported so far [47]. Although vmiRNAs encoded by RNA viruses are very rare, recently bovine leukemia virus has been found to encode a cluster of miRNAs transcribed by RNA Polymerase III, which is identical to human miR-29 [48]. Virus encoded miRNAs are also reported in culture cells as well as in cattle infected with bovine foamy virus (BFV), a member of retrovirus family and spumavirus subfamily [49]. Avian leukosis virus subgroup J (ALV-J), a member of avian retrovirus, also encodes a novel miRNA via canonical vmiRNA biosynthesis pathway. The vmiRNA has been designated as E (XSR) miRNA, since it is encoded by an virus specific region named exogenous element or XSR [50]. Similarly, *Torque teno viruses* (TTVs) a member of Anellovirus family also encodes miRNA, which inhibits the IFN signaling [51]. While studies of HIV-1 and Hepatitis B virus showed no direct evidence for vmiRNAs expression, computational analysis has predicted five pre-miRNAs in HIV-1 and one pre-miRNA in by Hepatitis B virus [52].

### miRNA modulating Hepatitis C virus infection

Considerable evidences suggesting the role of miRNAs in modulating Hepatitis C virus (HCV) life cycle, infectivity and host defense mechanisms have opened a novel avenue for innovative therapeutic approaches for HCV infection. miR-122, which is abundantly expressed in liver cells, interacts with HCV genomic RNA and facilitates its replication in infected cells [45, 53]. The interaction is mediated through binding of two copies of miR-122 to their respective seed map sites located within the 5′ UTR of the HCV genome [43, 45] The stable heterotrimeric interaction enhances HCV translation by promoting its association with ribosomes during the early initiation stage of translation [43]. Furthermore, miR-122 associated Argonaute proteins attached to the 5′ end of HCV genomic RNA protects the RNA from 5′ exonuclease activity, specifically of the 5′ to 3′ exoribonuclease 1 (Xrn1) [54, 55]. Interestingly, miR-122 interaction with the 5′ UTR of HCV RNA produces a 3′ overhang and masks the 5′ UTR, circumventing recognition by RNA helicase and ultimately reducing RNA decay [56]. Thus, miR-122 has a crucial role in enhancing HCV replication either by 5′ UTR masking or other mechanisms [57]. Targeting miR-122 could be a novel approach for developing a therapy against chronic HCV infections and the miRNA can be employed as a biomarker of hepatic damage by the virus. The most promising example for miRNA based therapeutic approach is miravirsen, an oligonucleotide which has been demonstrated to inhibit the function of miR-122 [43].

**Fig. 2:**
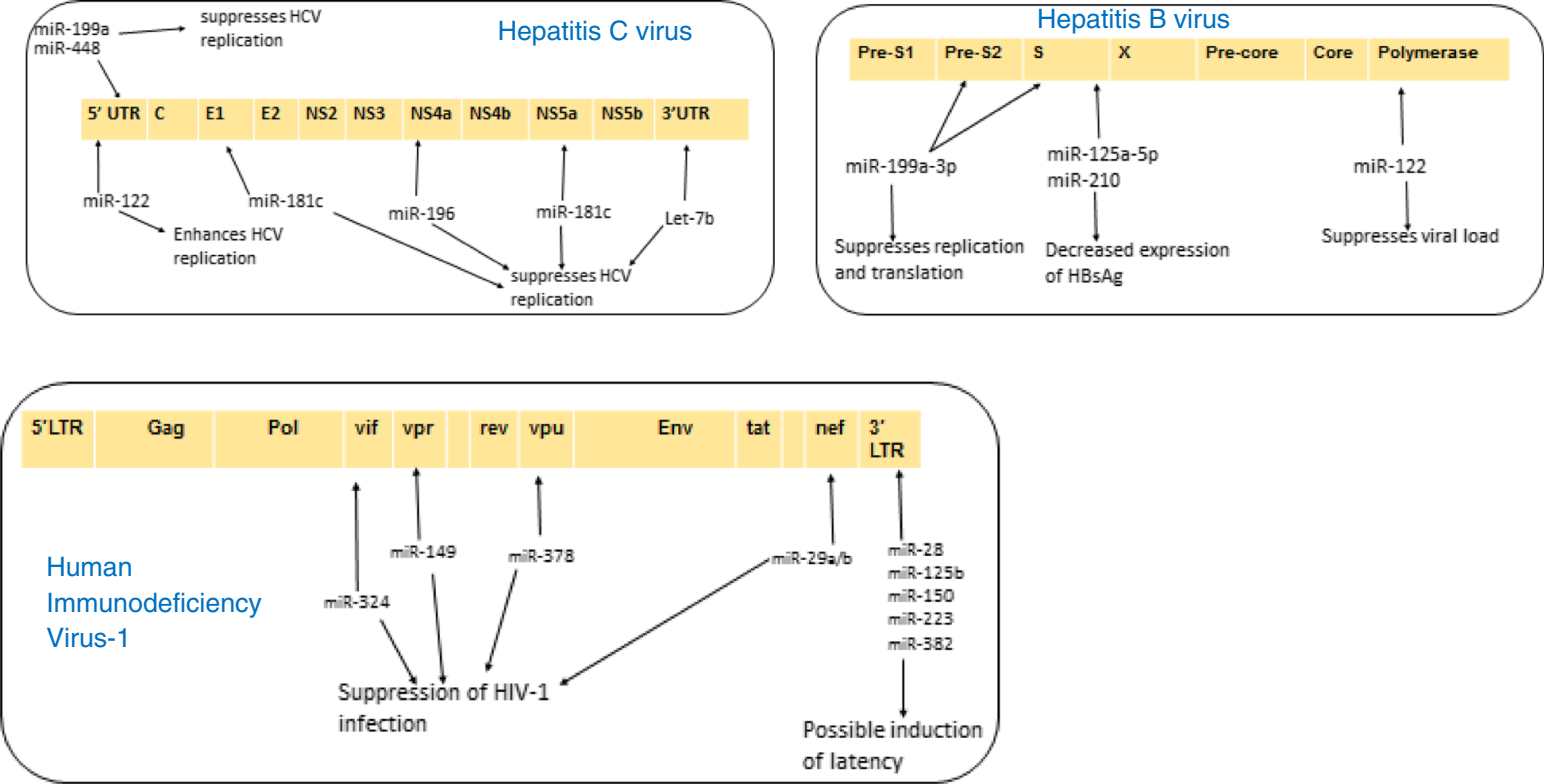
Some important miRNAs directly targeting the genomes of HCV, HBV and HIV-1 and their action

Conversely, miR-199a, Let-7b, miR-448 and miR-196 are all implicated in suppressing HCV RNA replication [58–60]. miR-199a counteracts the action of miR-122 and represses HCV replication by binding to the seed map site within the 5′ UTR of the HCV genome just downstream of the second miR-122 binding site [58]. Let-7b, expressed in various tissues including liver and spleen, binds to the HCV RNA genome at various positions including the 5′ UTR and NS5B coding region leading to repression of HCV replication, possibly inducing conformational changes in the viral RNA genome [59]. miR-196 and miR-448 are also capable of directly binding to and interacting with the HCV RNA genome and exerting inhibitory effects on HCV replication [60]. Recently, miR-181c was reported to bind to the E1 and NS5A regions of the HCV genome and have a down-regulating role in viral replication (Fig. 2) [8]. So, the alternative therapeutic approach could be upregulation of these miRNAs to suppress HCV replication.

In addition to the direct interaction with HCV RNA, some cellular miRNAs affect the HCV replication indirectly modulating replication-related pathways like Interferon (IFN)-mediated viral defense, Nuclear Factor-Kappa B (NF-κB), Hemeoxygenase 1 (HMOX1) and lipid metabolism (Table 1) [43]. Interestingly, HCV infection up-regulates miR-21 and miR-130a expression, both of which negatively regulate their target genes known to trigger viral replication in cells, by decreasing HCV-mediated IFN type I (IFN-I) production and disrupting the process of viral entry, respectively [61, 62]. Up-regulation of miR- 21, and some other miRNAs like miR-134, miR-320c, and miR-483-5p has been shown to inhibit the NF-κB and PI3K-Akt pathways thereby suppressing anti-viral effect in HCV-infected patients [63, 64]. Also, miR-196 suppresses the anti-viral effect on HCV by suppressing HMOX1 and miR-279 inhibits HCV replication through regulation of lipid metabolism [43].

**Table 1:** Summary of some important miRNAs modulating Hepatitis C and B and HIV-1 infection by targeting host factors

miRNAs	Targets	Actions
Hepatitis C virus		
miR-21	IFN1	Suppress viral replication
miR-130a	HCV entry	
miR-21/miR-134/miR-320c/miR-483-5p	NFkB and PI3K-Akt	Inhibit NFkB and PI3K-Akt signaling pathway
miR-196	HMOX1	Increase replication
miR-279	Lipid metabolism	Inhibit replication
miR-155	wnt signaling	Immune defense against the virus
Hepatitis B virus		
miR-122	upregulation of HMOX1	Decrease virus level in cell
miR-501	HBxIP	
miR-372/373	NFIB	Promotes replication
miR-155	IFN1	Suppress HBV disease pathogenesis
Human Immunodeficiency Virus type 1		
miR-27b	Cyclin T1	Prevent the activation of CD4+ cells
miR-155	TLR3/Lymphocytes/DC	Reduces HIV-1 infection
	3′ UTR of HDFs; LEDGF/p75, ADAM10, NUP 153	Decrease HIV replication
miR-146a	CXCR4	Prevents HIV-1 entry
miR-132	MeCP-2	Enhances HIV-1 infection
miR-217 and miR-34a	SIRT1	Enhances HIV-1 tat-mediated trans-activation
miR-182	NAMPT	Enhances HIV-1 tat-mediated trans-activation
miR-34a	PNUTS	Promotes HIV-1 transcription
miR-17/92 cluster and miR-20a	PCAF	Decrease susceptibility to HIV-1 infection

*DC* dendritic cells, *SIRT1* Sirtuin 1, *NAMPT* Nicotinamide phosphoribosyltransferase, *LEDGF* Lens Epithelium-derived Growth factor, *ADAM10* a disintegrin and metalloprotease, *MeCP2* methyl CpG binding protein 2, *HDF* HIV dependency factors, *nup153* Nuclear pore complex protein Nup153, HBx interacting protein, *NFkB* nuclear factor kappa B. *IFNI* Interferon I, *HMOX1* Heme oxygenase 1, *PNUTS* phosphatase 1 nuclear-targeting subunit, *PCAF* p300-CREB binding protein associated factor

In addition, some miRNAs are implicated in complications related to HCV infection although their target and exact mechanism of action is still not clear. Up-regulation of miR-276 promotes liver stenosis whereas down-regulation of miR-449a and miR-107, as well as up-regulation of miR-200c, supports hepatic fibrosis [43, 65]. Similarly, up-regulation of miR-155 and miR-141, as well as down-regulation of miR-152 and miR-491 (which are tumor suppressors), promotes hepatocellular carcinoma in HCV infection [43, 65]. miR-155, an indicator of hepatitis-induced hepatic damage, is over-expressed in the circulation of patients with chronic HCV infection [65]. Over-expression of miR-155 may inhibit apoptosis of hepatocytes and promote cell proliferation by activating Wnt signaling leading to progression to hepatocellular carcinoma [65, 66]. Conversely, the suppression of miR-155 may lead to cell cycle arrest in the G0 or G1 phase suggesting the positive role of miR-155 in immune defense against HCV infection [65]. Therefore, balanced expression of miR-155 is essential to retard the development of hepatocellular carcinoma and at the same time promote adequate cell cycle.

### miRNA modulating Hepatitis B virus infection

Several cellular miRNAs modulate Hepatitis B virus (HBV) replication directly by binding to its transcripts (Fig. 2) or indirectly by targeting cellular factors, genes and signaling pathways related to HBV replication and pathogenesis (Table 1) [67]. The miRNAs shown to reduce HBV replication and the expression of HBV surface antigen (HBsAg) are miR-199a-3p, miR-210 and miR-125a-5p. Among them, miR-199a-3p targets HBsAg coding region and the pre-S region of the HBV genome, whereas miR-125a-5p binds to HBsAg mRNA leading to inhibition of its translation [46, 68]. miR-122 which enhances HCV replication, has opposite effect on HBV, where it upregulates HMOX1 to reduce HBV levels in cells. miR-122 also has cyclin G1-modulated anti-HBV activity and directly affect the viral DNA polymerase [46]. These findings imply that Miravirsen targeting miR-122 will have opposite effect if used as therapeutics and other candidate miRNAs need to be considered.

Conversely, HBV can trigger changes in the expression of cellular miRNAs targeting negative regulators of HBV replication [69]. miR-501 which targets HBx interacting protein (HBxIP) and miR-372/373 which targets nuclear factor I B (NF-IB) of the host to promote viral replication are upregulated by HBV [70, 71]. HBx protein of HBV supports cellular proliferation by downregulating let-7a, which negatively regulates cellular proliferation partly through targeting signal transducer and activator of transcription 3 (STAT3) [69]. Furthermore, some miRNAs are involved in induction and suppression of HBV-related complications like hepatocellular carcinoma and cirrhosis. miR-155 by up-regulation of IFN-inducible genes suppresses HBV disease progression, whereas miR-548 by down-regulation of the host anti-viral response promotes HBV progression [46].

### miRNA modulating Human Immunodeficiency Virus infection

Cellular miRNAs have been shown to restrict Human immunodeficiency virus type 1 (HIV-1) infection either by direct binding to the viral RNA or by indirect modulation of HIV Dependency Factors (HDFs) (reveiwed in ref [72]). CD4^+^ T-cells have been found to express some miRNAs which target mRNA of the viral genes (Fig. 2); negative regulatory factor (nef), viral protein s(vpr), viral infectivity factor (vif) and viral protein U (vpu); and regulate host factors like Cyclin T1 protein and receptors or co-receptors needed for HIV entry [73–76]. Similarly other cells involved in the spread of HIV-1 within the body are also reported to express some miRNAs that defend against the virus [30].

The miRNAs that directly bind to the 3′UTR of HIV-1 RNA (Fig. 2) include miR-28, miR-125b, miR-150, miR-223 and miR-382, which are highly expressed in resting CD4+T cells [73]. Activation of resting CD4^+^ T-cells (which results in more HIV replication) leads to down-regulation of these miRNAs, which can be correlated with enhanced susceptibility to HIV-1 infection [73]. Other miRNAs target host factors and indirectly modulate HIV-1 replication, susceptibility and latency in different cells (Table 1). miR-27b has been reported to inhibit the expression of Cyclin T1 in resting CD4^+^ T-cells. Activation of CD4+ T-cells has been shown to down-regulate miR-27b, subsequently up-regulating Cyclin T1. Up-regulation of Cyclin T1 results in enhanced HIV-1 susceptibility of CD4^+^ T-cells [77]. Also, miR-155 has been shown to regulate immune responses to HIV-1 infection by altering lymphocyte responses, inhibiting dendritic cell binding to HIV-1 and modulating TLR3 stimulation in macrophages leading to anti-HIV-1 effects [65]. miR-155 has been reported to target the 3′ UTR of mRNAs of the HDFs; the lens epithelium-derived growth factor (LEDGF)/p75, ADAM10 (a disintegrin and metalloprotease 10) and nucleoporin (NUP153), and down-regulate their protein expression in primary macrophages [26]. Opposing the findings described above, Cullin et al. reported that HIV-1 neither encodes viral miRNAs nor strongly modulates cellular miRNA expression possibly due to extensive RNA secondary structures that block potential miRNA binding sites [78].

The miR-17/92 cluster and miR-20a are reported to target p300-CREB binding protein associated factor (PCAF) and make the cell less susceptible to HIV-1 infection, because PCAF has a role in tat acetylation (acetylated tat is transcriptionally more active) [79]. On the other hand, HIV-1 tends to suppress these miRNAs to upregulate PCAF expression and possibly increasing viral infectivity [80]. It has -been reported that expression of purine-rich element binding protein alpha (Pur-α), which facilitates HIV-1 transcription by binding tat protein and the TAR element, was significantly lower in monocytes than in monocyte-derived dendritic cells (DCs) [81]. Several of the cellular miRNAs (miR-15a, miR-15b, miR-16, miR-20a, miR-93 and miR-106b) which target pur-α mRNA were overexpressed in monocytes. The correlation between low expression of pur-α and overexpression of these miRNAs indicates their role in decreasing susceptibility to HIV-1 infection [80, 82].

The interaction between HIV-1 RNA and proteins involved in the biogenesis of cellular miRNAs has also been reported. Silencing the genes for Drosha and Dicer showed increased viral replication in HIV-1-infected mononuclear cells even with latent infection [80]. Furthermore, HIV-1 gene expression was found to be negatively regulated by Argonaute or other miRNA related proteins by restricting the association of viral mRNA with polysomes [30]. Interestingly, silencing of Dicer has led to virus re-activation in peripheral blood mononuclear cells (PBMCs) isolated from HIV-1-infected patients undergoing suppressive combinational antiretroviral therapy (cART) [30].

On the other hand, some cellular miRNAs enhance HIV-1 replication by various mechanism. Over-expression of miR-132 down-regulates methyl CpG binding protein 2 (MeCP2) leading to enhancement of HIV-1 infection [76]. In addition, miR-217 and miR-34a, which have been reported to be up-regulated by tat exposure, bind to the 3′ UTR of Sirtuin 1 (SIRT1) mRNA inhibiting its expression. Inhibition of SIRT1 which de-acetylates tat protein and regulates HIV-1 transcription, enhances HIV-1 tat-mediated transactivation [83, 84]. Furthermore, a recent study revealed that down-regulation of nicotinamide phosphoribosyl transferase (NAMPT) by miR-182 led to decreased SIRT1 expression levels, which in turn enhanced HIV-1 Tat trans-activation [30, 72]. HIV-1 induces the expression of miR-34a, which promotes HIV-1 replication in CD4+ T cells. The other target of miR-34a is phosphatase 1 nuclear-targeting subunit (PNUTS), which negatively regulates HIV-1 transcription by inhibiting the assembly of cyclin T1 and CDK9 to block the transcription elongation [85].

### Role of miRNA in Dengue virus infection

A group of researchers has reported that Dengue virus (DENV) infection down regulates host cellular miRNA elements, notably Dicer, Drosha, Ago1 and Ago2, to facilitate its replication. They found that the downregulation was mediated by interferon independent activity of the viral protein NS4B which interferes the dicing of precursor miRNA [86]. DENV infection significantly induces the expression of miR-146a, which facilitates viral replication by targeting TNF Receptor Associated Factor 6 (TRAF6) and dampening Interferon β (IFN-β) production [87]. All four subtypes of DENV downregulate the miRNA-133a which suppresses viral replication possibly via interference with polypyrimidine tract binding protein (PTB) expression [88]. Yet, another miRNA namely miR-548g-3p also regulates the replication of DENV by targeting stem loop A (SLA) promoter element within the 5′UTR of DENV genome [89]. miRNA expression profiling followed by qRT-PCR validation has revealed that 4 miRNAs were upregulated and 2 were downregulated in dengue patients. miR-21-5p and miR-146a-5p, which were functionally involved in inflammation and cell proliferation, were significantly different from the control groups indicating their high sensitivity and specificity as indicators of dengue infection. Further, these two miRNAs were correlated with number of leucocytes and neutrophils. These findings have suggested that miRNAs can also be employed as a diagnostic marker for DENV infection [90]. On the other hand, small viral RNA resembling miRNA found in mosquitos infected with DENV type 2 is reported to specifically target a viral gene and auto-regulates viral replication [91]. Incorporation of the miRNA recognition element (MRE) of miR-122 into the viral mRNA might have inhibitory effect on susceptibility to DENV infection in hepatic cells, rendering the virus safer for vaccine preparation [92].

### Role of miRNA in Herpes virus infections

Most of the miRNAs encoded by *Herpes simplex virus*-*1* (HSV-1) and *Herpes simplex virus-2* (HSV-2) are expressed in latency and bind to latency-associated transcripts, underscoring their role in the development of latency during Herpes virus infection. The principal target for the latency-inducing miRNAs (e.g. miR-138) is the infected cell polypeptide 0 protein (ICP0), which induces lytic genes in neuron [93]. Similarly, host cell miR-155, miR-146a and miR-21 are up-regulated in B-cells when latently infected by *Epstein Bar Virus* (EBV) [94]. miR-155 mediated attenuation of NF-κB signaling in B lymphocytes by EBV is pivotal for the stable maintenance of EBV genome and immortalization of B lymphocytes during latency [95]. Conversely, EBV latent membrane protein 1 (LMP1) upregulates miR-155 expression by activation of NF-kB pathway [96]. So, the complex interaction among miR-155, LMP1 and NF-kB signaling seems to control the immortalization and latency in EBV infection. *Cytomegalovirus* (CMV) infection has been reported to repress the expression of miR-100 and miR-101, which are linked to apoptosis [97]. Some oncogenic herpesviruses encode viral miRNA that mimic and exploit miR-155-mediated regulatory pathways for their own benefit. Kshv-miR-K12-11, the viral functional ortholog of miR-155 encoded by *Kaposi sarcoma-associated herpesvirus* (KSHV), mimics miR-155 and utilizes its regulatory pathway to immortalize infected lymphocytes. KSHV-miR-12-11 attenuates TGF-β via downregulation of SMAD5 [98]. The other miR-155 functional ortholog, miR-M4 encoded by *Marek’s disease chicken virus* (MDV), may also be responsible for contributing to viral oncogenicity by down-regulating its target latent TGF-β-binding protein-1 (LTBP1), leading to reduced secretion of TGF-β1. The resulting suppression of TGF-β signaling might contribute to the activation of the oncogene c-Myc [65].

### Viral RNAi suppressors

Viral RNAi suppressors (VSRs) are the virus encoded proteins that counteract against the RNA interference mediated by miRNA or other small RNA. Extensive research has been carried out in plant and insect VSRs, but only a few of them from mammalian viruses have been identified [99]. Some of the examples for mammalian VSRs are nucleocapsid protein (N protein) of *Corona viruses*, VP35 protein from Ebola *virus*, tat protein of HIV, Core protein (C) of HCV, nonstructural protein 1 (NS1) from Human influenza virus and B2 protein of *Nodamura virus* [100–104]. N protein of *Corona viruses*, via its double-stranded RNA binding activity, suppresses the short hairpin RNAs (shRNAs) or siRNAs from mammalian cells [100]. Similarly, VP35 protein from Ebola virus, which is an antagonist of IFN, suppresses RNAi interference by binding to dsRNA in human cells [101]. Whereas, HIV-1 tat and HCV core protein counteract the RNAi in human cell lines by downregulating the expression or activity of Dicer, which processes precursor hairpin structures [102, 103]. Function of tat as a suppressor of RNAi by suppressing the common pathway in small RNA maturation is conserved across plants as well as animals including human [105]. A novel viral motif, arginine rich motif (ARM) in the HIV-1 tat and rev has been shown to counteract the RNA interference mediated by Dicer [106].

Similarly, HIV-1 vpr has also been reported to alter the expression of Dicer. Trans-activating response element (TAR) of the HIV-1 transcript and miRNA were reported to be packaged in exosomes released from HIV-1-infected cells. The miRNA taken together with TAR might modulate uninfected cells, perhaps to increase their susceptibility to infection [30]. Contrastingly, HIV-1 tat, HTLV-1 Tax and BFV Tas are not capable of suppressing the RNA interference either by siRNA or miRNA in human cells [107]. NS1 protein, one of the virulent factor of *Influenza virus A*, has also demonstrated its RNAi suppressor activity in plants and animal cells [108]. In *Nodamura virus*, a small virus infecting mammalian as well as insect host, B2 protein binds to Dicer and prevent post Dicer activity of RNAi [109]. Yet another mammalian virus, *Porcine Reproductive and Respiratory Syndrome Virus (PRRSV*), has also been reported to suppress shRNA, dsRNA and miRNA mediated RNA interference. Furthermore, this virus also down regulates the expression of Ago-2 [104].

### Epigenetic control of miRNA by pathogens

Many of the miRNA such as miR-9, miR-34, miR-137, miR-148, miR-124; are found to be controlled by epigenetic mechanism like DNA methylation and histone acetylation [110]. Conversely, some of the miRNAs modulate the epigenetics by controlling the epigenetic regulators such as methyl transferase, histone deacetylases and polycomb group genes [111]. Epigenetic changes of miRNA or tumor suppressor genes can be induced by pathogenic microorganism, leading to carcinogenesis. Aberrant methylation of miRNA genes (hsa-miR-124) in cervical cancer cell line caused by *Human papilloma virus* (HPV) has also been reported [112, 113]. On the other hand, *Cryptosporidium parvum*, a protozoal parasite, hijacks the histone deacetylase and NF-κB signaling pathway to suppress two host miRNA namely miR-424 and miR-503 in the epithelial cells [111]. Two of the miRNA namely miR-1 and miR-152 are reported to be involved in HBV pathogenesis via epigenetic control [114, 115]. miR-1 upregulates HBV transcription by enhancing farnesoid X receptor α expression but downregulates host cell cycle and cell proliferation by targeting histone deacetylase 4 and E2F transcription factor 5 [114]. Whereas, miR-152 reduces the levels of DNA methyltransferase 1 (DNMT1) in HBV induced Hepatocellular carcinoma by targeting 3′UTR of DNMT1 [115].

## Conclusion and perspectives

Cellular miRNAs are important components of the host defense mechanism against viral infections. Many viruses are able to modulate cellular miRNA expression in host cells mostly in order to facilitate their survival, replication and pathogenesis. But, the overexpression of miRNA triggered by pathogens is not always correlated with their survival or pathogenesis and is sometimes cell or tissue specific. The exact mechanism(s) of modulation of host cellular miRNA by viruses and specific virulence factors is still unclear. Unraveling the molecular mechanism(s) of miRNA modulation by viral infections and vice versa will give direction to novel therapeutic approaches. Manipulation of miRNA, either by miRNA analogs or by inhibitors, could be a novel approach for developing therapies and prophylactic vaccines for various life-threatening viral infections. In addition, miRNAs may be exploited as biomarkers for laboratory diagnosis and prognosis. Some of the important cellular miRNA implicated in some viral infections are summarized in Table 1.

Some viruses encode for virus-specific miRNAs which are expressed in the host cell to subvert host defense and allow intracellular persistence. Understanding the specific functions of viral- miRNAs in the host-pathogen relationship will be another important step for targeting these miRNAs. All in all, miRNA may be a tool for diagnostic and therapeutic innovations against viral infections.

## Abbreviations

HBV: Hepatitis B virus
HCV: Hepatitis C virus
HDF: HIV dependency factor
HIV: Human Immunodeficiency Virus
HMOX1: Hemeoxygenase 1
IFN: Interferon
lncRNA: Long non-coding RNA
miR: microRNA
miRISC: microRNA-induced silencing complex
mRNA: Messenger RNA
NAMPT: Nicotinamide phosphoribosyl transferase
NF-kB: Nuclear Factor Kappa B
PCAF: p300-CREB binding protein associated factor
Pri-miRNA: Primary miRNA
Pur-α: Purine-rich element binding protein alpha
RBP: RNA binding protein
RNA: Ribonucleic acid
SIRT1: Sirtuin 1
sncRNA: Small non-coding RNA
SRS: Suppressor of RNA silencing
TAR: Transactivation Response RNA
TLBP-1: TGF-β-binding protein-1
TLR: Toll-like receptor
UTR: Untranslated region
